# Hypoxia Induced Sex-Difference in Zebrafish Brain Proteome Profile Reveals the Crucial Role of H3K9me3 in Recovery From Acute Hypoxia

**DOI:** 10.3389/fgene.2021.635904

**Published:** 2022-01-31

**Authors:** Tapatee Das, Avijeet Kamle, Arvind Kumar, Sumana Chakravarty

**Affiliations:** ^1^ Applied Biology, CSIR-Indian Institute of Chemical Technology (IICT), Hyderabad, India; ^2^ Academy of Scientific and Innovative Research (AcSIR), Ghaziabad, India; ^3^ CSIR-Centre for Cellular and Molecular Biology (CCMB), Hyderabad, India

**Keywords:** sex difference, IPA, pathway analysis, iTRAQ, hypoxia-ischemia recovery

## Abstract

Understanding the molecular basis of sex differences in neural response to acute hypoxic insult has profound implications for the effective prevention and treatment of ischemic stroke. Global hypoxic-ischemic induced neural damage has been studied recently under well-controlled, non-invasive, reproducible conditions using a zebrafish model. Our earlier report on sex difference in global acute hypoxia-induced neural damage and recovery in zebrafish prompted us to conduct a comprehensive study on the mechanisms underlying the recovery. An omics approach for studying quantitative changes in brain proteome upon hypoxia insult following recovery was undertaken using iTRAQ-based LC-MS/MS approach. The results shed light on the altered expression of many regulatory proteins in the zebrafish brain upon acute hypoxia following recovery. The sex difference in differentially expressed proteins along with the proteins expressed in a uniform direction in both the sexes was studied. Core expression analysis by Ingenuity Pathway Analysis (IPA) showed a distinct sex difference in the disease function heatmap. Most of the upstream regulators obtained through IPA were validated at the transcriptional level. Translational upregulation of H3K9me3 in males led us to elucidate the mechanism of recovery by confirming transcriptional targets through ChIP-qPCR. The upregulation of H3K9me3 level in males at 4 h post-hypoxia appears to affect the early neurogenic markers nestin, klf4, and sox2, which might explain the late recovery in males, compared to females. Acute hypoxia-induced sex-specific comparison of brain proteome led us to reveal many differentially expressed proteins, which can be further studied for the development of novel targets for better therapeutic strategy.

## Highlights

⁃ Sex disparity was observed in differentially regulated proteins; mostly downregulated in males.
⁃ Five common transcription regulators [Myc, Mknk1, Nfe2l2 (Nrf2), Thrb, and Otx 2] have differential activation states.
⁃ Upon CoIP, H3K9me3 targets of hypoxia were found to be totally different from normoxia.
⁃ H3K9me3 seems to be a key player in early neurogenesis.
⁃ Novel finding: H3K9me3 appeals to play an important role in the delayed recovery of males from acute hypoxia.

## Introduction

Oxygenation in vertebrates is always a life-or-death necessity for any of the metabolic needs of cells and tissues (Sun, 1999). Over the last decade, we have acquired adequate information on cellular and molecular mechanisms in hypoxic-ischemic injury, survival, and death (Muller and Marks, 2014; Sekhon et al., 2017). Hypoxic-ischemic neural injury continues to be the leading cause of death and disability worldwide (Catherine and Collaborators, 2019). The degree of disability does not simply reflect the severity or distribution of the impaired blood supply (Dugan et al., 1999). The most common condition of hypoxia-ischemia leads to cerebral stroke due to the focal disruption of blood supply to a part of the brain. Other conditions include transient impairment of blood flow to the entire brain, termed global ischemia, which occurs following cardiac arrest.

A low level of oxygen and the brain’s susceptibility to acute hypoxia characterizes the key factor determining critical dependency. Cerebral oxygenation is reduced in hypoxia and neuronal damage can occur during a prolonged mismatch between oxygen supply and demand (Goodall et al., 2014). All the neurons in the brain can sense and, crucially, modify, their activity in response to hypoxia. Most neurons respond to hypoxia by decreasing metabolic demand and thus the need for aerobic energy (Michiels, 2004). Deciphering cellular response to energetic challenges that occur on the onset of acute hypoxia may give insight into the ischemic condition in various diseases (Bickler and Buck, 2007). Broad high throughput approaches in global changes in protein expression allow uncovering the critical signals underlying mechanisms in the disease condition. Acute Hypoxia causes a significant perturbation in cellular energy homeostasis before a hypoxia sensing and signal transduction cascade needing energy demand initiates (Hochachka et al., 1996). An early component of the responses to acute hypoxia i.e., neural damage and recovery may have both post-transcriptional and translational mechanisms. The rapid response to acute hypoxia may preclude many pathways that require many new gene expressions suggesting the mechanism underlying recovery from acute hypoxia is mediated at least in part by the activities of the existing pool of mRNA and protein. An approach such as high throughput proteomic analysis is one of the ideally suited approaches to understand the neural changes induced by acute hypoxia with recovery (Li et al., 2019).

Previous proteomic studies have shown hypoxia-induced changes in the zebrafish (*Danio rerio*) skeletal muscle proteome (Chen et al., 2013) and have implicated a broad range of cellular functions in response to hypoxia. Another proteomics study on zebrafish brain upon chronic unpredictable stress (Chakravarty et al., 2013) has recently laid the groundwork for the analysis of neural proteome response to stressors.

A recent review article on Proteomics-Based Approaches for the Study of Ischemic Stroke (Li et al., 2019) discussed the proteomics study of ischemic stroke using *in vivo* and *in vitro* models, with and without interventions and taking tissue, cerebrospinal fluid, or plasma. Although proteomic studies have contributed with a long list of potential biomarkers for diagnosis, prognosis, and monitoring of ischemic stroke, most of these have not been implemented in clinical application successfully. The shortcomings from the existing proteomics data are small sample size, cell types, te age of experimental animals, and using single-sex experimental animals all seem to be responsible for blocking these results from achieving clinical implementation.

Like many neurological disorders, cerebral stroke is reported to have sex-specific differences in occurrence and mechanisms. However, the molecular details underlying these sex-specific differences have not yet been explored using a relevant animal model. In fact, many factors including genetics, hormones (estrogen and androgen), epigenetic regulation, and environment contribute to sex-specific differences. Since ischemic sensitivity varies over the lifespan, and the “ischemia resistant” female phenotype diminishes after menopause, hence the role of sex hormones cannot be ruled out. To understand the role of hormonal status on the cerebral vasculature in pinpointing sex-specific differences in stroke pathophysiology, a suitable, simple animal model that can help to address these complicated sex-specific differences is warranted.

Sex-specific differences in the hypoxic-ischemic brain have profound implications for effective prevention and treatment. Global hypoxic-ischemic damages and recovery are well studied under the well-controlled, non-invasive, reproducible conditions in zebrafish (Yu and Li, 2013; Braga et al., 2016; Silva et al., 2016; Das et al., 2019). In our previous study, we have reported the sex-specific difference in hypoxia-induced neural damage and recovery, where we have concluded that as compared to males, females showed a higher level of neural damage and an ability to recover faster. This interesting finding led us to explore the global proteome changes induced in recovery after the hypoxic stress, so in the present study, we performed a high throughput proteomic analysis on zebrafish brain by iTRAQ method. The iTRAQ labeling method also allows the identification of different post-translational modifications which are key to understand the aetiology and develop better treatment.

## Experimental Procedure

### Animal Procurement and Acute-Hypoxia Treatment

Wild type strain of zebrafish was bred and raised at CSIR-IICT zebrafish facility in accordance with protocol no IICT/CB/SC/281114/30 under registration no# 97/1999/CPCSEA. All the experimental animals were maintained in a controlled environment with a 14 h light/10 h dark cycle at 28°C with three feedings and constant aeration. Zebrafish aged 5–6 month were segregated on the basis of sex and used for all the experiments. For an acute hypoxia treatment animals were placed in an air-tight glass hypoxia chamber for a period of 5 min with 0.6 mg/ltr dissolved oxygen following reoxygenation at 7 mg/ltr dissolved oxygen in a recovery tank, which is exactly described in (Das et al., 2019). After 4 h post-hypoxia, all the animals were sacrificed for brain tissue collection.

### Protein Extraction for iTRAQ

The animals were euthanatized and decapitated to remove the brain. The whole brain from each animal was homogenized in a lysis buffer [50 mM ammonium bicarbonate pH 8.0, 0.1% SDS with protease inhibitor cocktail (Sigma)] and for further efficient disruption and homogenization of tissue, a mild sonication was done using Bioruptor^®^. The obtained lysates were cold-centrifuged at 14,000 rpm for 15 min and the supernatant was quantified using Bradford assay with BSA as standard. Further protein samples were cleaned up by acetone precipitation. For each group, 80 µg of protein was taken and six volumes of chilled acetone were added for precipitation. After decantation of acetone the samples were resuspended in dissolution buffer (Buffer pH is 8.5. Contains 0.5 M triethyammonium bicarbonate) provided with the iTRAQ^®^ Reagents-4plex Applications kit-Protein (AB Sciex). Before trypsin digestion, all the protein samples were reduced and cysteine blocked using the reagents provided in the iTRAQ^®^ Reagents-4plex Applications kit-Protein (AB Sciex). Digestion and labeling of proteins were done according to the manufacturer’s protocol. The samples from normoxia male and female were labeled with reagents 114 and 116 and the samples from hypoxia male and female were labeled with reagents 115 and 117, respectively. Subsequently, all the labeled samples were pooled and vacuum dried, and further cleaned up using the C18 desalting column (Thermo Fisher Scientific). The final fraction was concentrated using a vacuum concentrator and reconstituted in 10 µl of 0.1% formic acid for LC-MS/MS analysis.

### LC-MS/MS Analysis

LC-MS/MS analysis of the trypsin digested iTRAQ labeled and purified fractions were performed in LTQ - Orbitrap Velos (Thermo Scientific, Germany). The fragmentation was carried out using higher-energy collision dissociation (HCD) with 50% normalized collision energy. The MS data were analyzed using Proteome Discoverer (Thermo Fisher Scientific, Version 1.4). MS/MS search was carried out using the SEQUEST search engine against the NCBI zebrafish protein database. Search parameters included trypsin as an enzyme with a maximum of two missed cleavage allowed; precursor and fragment mass tolerance were set to 10 ppm and 0.2 Da respectively; Methionine oxidation was set as a dynamic modification while methylthio modification at cysteine and iTRAQ modification at N-terminus of the peptide were set as static modifications. The FDR was calculated by enabling the peptide sequence analysis using a decoy database. High confidence peptide identifications were obtained by setting a target FDR threshold of 1% at the peptide level. Relative quantitation of proteins was determined based on the ratios of relative intensities of the reporter ions from hypoxia treated and untreated samples released during MS/MS fragmentation of each peptide. Appropriate quality control filters at the level of peptides/peptide spectral matches (PSMs) and then at the protein level were applied to the iTRAQ data. Proteins identified from the triplicate runs as having more than 1.5-log-fold changes in the hypoxia samples against the normoxia samples were selected for upregulation and having less than 0.5-log fold change considered to be downregulated for its differential expression. Proteins based on their regulation were analyzed for putative associations in different network pathways.

The mass spectrometry proteomics data have been deposited to the ProteomeXchange Consortium via the PRIDE partner repository with the dataset identifier PXD027528".

### Protein Enrichment Analysis

To perform the functional enrichment tests of the candidate proteins, we used Ingenuity Pathway Analysis (IPA) software for both canonical pathways and molecular networks altered. The IPA system provides a more comprehensive pathway resource based on manual collection and curation. The rich information returned by IPA is also suitable for pathway crosstalk analysis as it has more molecules and their connections included. For analysis, we have provided the identified peptides with relative and absolute expression fold change values and performed core IPA analysis, biomarkers, and molecular and functional comparison analysis.

### Co-Immunoprecipitation

Zebrafish brain tissue was homogenized in nuclear extraction buffer [50 mM HEPES (pH 7.8), 50 mM KCl, 300 mM NaCl, 0.1 M EDTA, 1 mM DTT, 10% (v/v) Glycerol and 1X protease inhibitor] and further washed with PBS and incubated in RIPA buffer [20 mM Tris (pH 7.5), 150 mM NaCl, 1% NP-40, 5 mM EDTA, protease and phosphatase inhibitors] for 15 min on ice. After centrifugation, the supernatant was collected and pre-cleared with protein A agarose beads (Santa Cruz) at 4°C for 30 min. The pre-cleared lysate was then incubated with Anti-Histone H3 (tri methyl K9) antibody (H3K9me3) (AB8898 1:250) complexed to protein A beads at 4°C for 5–6 h, followed by washes with a buffer containing 10 mM Tris (pH 7.5), 150 mM NaCl, and 1 mM EDTA. The beads complexed with the immunoprecipitated proteins were then boiled at 100°C in 3X Laemmli buffer for 5 min. 2.5% of whole tissue lysate was taken as input for each immunoprecipitation. Western blotting was carried out by loading equal amounts of the immunoprecipitated proteins.

### Immunoblotting Analysis

For immunoblotting experiments, cells were lysed in 3X Laemmli buffer [180 mM Tris (pH 6.8), 6% SDS, 15% glycerol, 7.5% β-mercaptoethanol, and 0.01% bromophenol blue]. Images were captured using Chemicapt (Vilber-Lourmat, Germany). Densitometry analysis for blots was performed using ImageJ software (NIH) and images were processed in Adobe Photoshop CS3. The intensity values plotted or mentioned are average values from the number of biological replicates indicated in the legend.

### Chromatin Immunoprecipitation Assay

ChIP was performed as described in (Weidemann et al., 2013) with required minor modifications. Briefly, for each ChIP, cross-linked samples from three animals were pooled together both in the normoxia and hypoxia groups. The 30 μg of chromatin from each sample was pre-cleared with Dynabeads (Invitrogen) before incubation with an anti-rabbit H3K9me3 antibody (EPR16601) keeping non-immune rabbit IgG antibody as a negative control. After reverse cross-linking and sequential washes with different concentration salt buffers, DNA was purified using phenol-chloroform-isoamyl alcohol (25:24:1 ratio, SIGMA). Specific primers for the gene-specific 5′ upstream region of the transcription start site were used for quantifying the enrichment of the histone mark H3K9me3, for 10% input, in SYBR Green-based qPCR assays.

### qPCR

The total RNA was isolated using TRIzol Reagent as per the manufacturer’s instruction. The cDNA was synthesized employing RevertAid H Minus First Strand cDNA Synthesis Kit according to the manufacturer’s protocol. The primer sequences are available on a request basis. Real-time PCR was performed in triplicate using SYBR Green PCR Master Mix Detection System (Applied Biosystems). Normalization of mRNA expression levels was carried out using β-actin as the housekeeping gene. Gene expression was normalized against the ubiquitously expressed beta actin gene. Data were analyzed using the Δ(ΔCT) method.

### Statistical Analysis

Statistical analysis was performed using Microsoft Excel. Mean differences between the normoxia and hypoxia groups were determined using a two-tailed unpaired Student’s *t*-test with confidence intervals of 95% since only two groups were used to compare a single variable i.e., normoxia/hypoxia. A *p*-value of ≤0.05 was considered significant.

## Results and Discussion

### Analysis of Zebrafish Brain Proteome Induced by Acute Hypoxia and During Recovery Using iTRAQ Based LC-MS/MS

**FIGURE 1:**
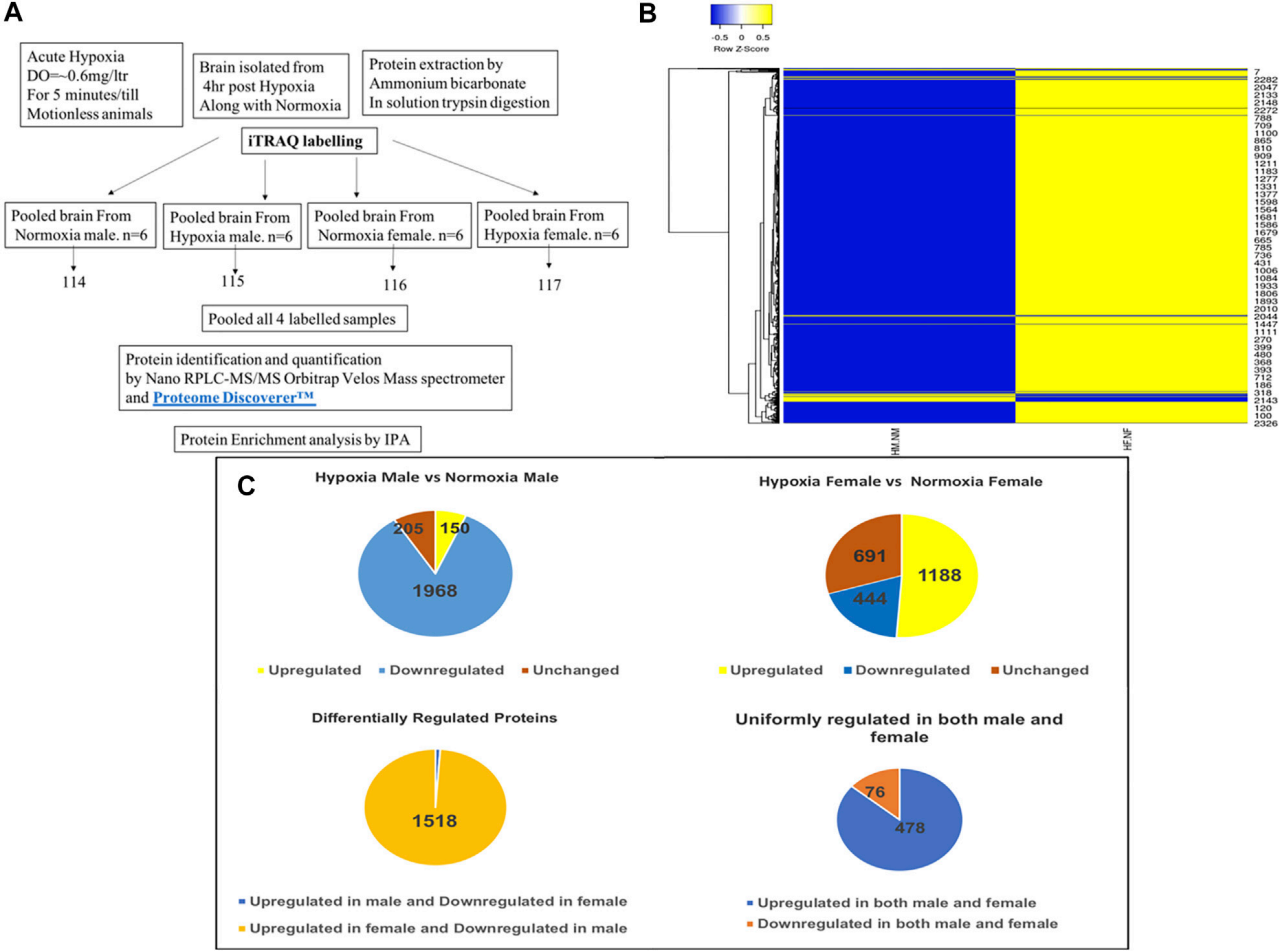
Analysis of zebrafish brain proteome by iTRAQ. Schematic representation of brain proteome analysis by iTRAQ labeling **(A)**, Cluster heatmap of proteins obtained from iTRAQ analysis for hypoxia male [HM] vs. normoxia male [NM] and hypoxia female [HF] vs. normoxia female [NF] **(B)**, Pie chart showing the analysis of expression of proteins resulted from iTRAQ in hypoxia male and female brain compared with normoxia male and female brain **(C)**.

As described previously by us (Das et al., 2019), 4 h post-acute hypoxia treatment (for 5 min at DO = ±0.6 mg/litre) the brain tissues from both hypoxia-treated and untreated male and female zebrafish were isolated (*n* = 6 per group) and subjected to comprehensive proteomic profiling. For this, male and female zebrafish brains of nine individuals from each sex were pooled together for iTRAQ based LC-MS/MS method and three technical replicates were run in LC-MS/MS, as depicted in Figure 1A. Differentially expressed proteins were identified using iTRAQ and LC-MS/MS analysis on an LTQ Orbitrap Velos mass spectrometer, by comparing hypoxia male (HM) vs. normoxia male (NM) and hypoxia female (HF) vs. normoxia female (NF) (Figure 1A). A total of 2,323 proteins were identified to be regulated differentially after the data analysis from experimental runs in triplicate. The resuling proteins (2,323) were used to generate a clustered heatmap for showing the sex difference in expression patterns (Figure 1B). A majority (i.e., 84%) of differentially regulated proteins, 1,968 in total, were found downregulated in HM versus NM. In contrast, more than half (51%) of differentially regulated proteins, 1,188 in total, were found upregulated in HF versus NF (Figure 1C). 1,535 proteins were differentially regulated, among those 1,518 (98%) proteins were upregulated in females and downregulated in males whereas only 17 (2%) proteins were upregulated in males and downregulated in females, showing a clear differential regulation in a sex-specific manner. Another 554 proteins showed a similar expression pattern in both sexes, where 478 proteins were upregulated and 76 proteins downregulated. A list of differentially regulated proteins is provided in Table 1.

**TABLE 1:** IPA generated disease function analysis for zebrafish male and female brain proteome induced by hypoxia following recovery.

**Male ZF brain proteome analysis (HM/NM)**					
Categories	Diseases or functions annotation	*p*-value	Predicted activation state	Activation z-score	# Molecules
Organismal survival	Organismal death	2.31E-11	Increased	12.434	251
Cell death and survival	Cell death	2.51E-11	Increased	4.843	218
Cell death and survival	Necrosis	3.27E-09	Increased	4.131	154
Cancer, organismal injury and abnormalities, respiratory disease	Development of lung tumor	9.13E-08	Increased	3.302	27
Cancer, organismal injury and abnormalities	Incidence of tumor	0.000000893	Increased	2.199	87
Cell death and survival	Apoptosis	0.000000991	Increased	4.38	153
Cancer, organismal injury and abnormalities	Malignant genitourinary solid tumor	0.00000154	Increased	2.272	33
Cancer, organismal injury and abnormalities	Frequency of tumor	0.00000449	Increased	2.133	77
Cancer, organismal injury and abnormalities, respiratory disease	Lung carcinoma	0.0000213	Increased	2.584	23
Cancer, organismal injury and abnormalities	Tumorigenesis of epithelial neoplasm	0.0000284	Increased	2.18	52
Cancer, organismal injury and abnormalities	Development of adenocarcinoma	0.0000307	Increased	2.525	28
Cell death and survival	Apoptosis of neurons	0.0000414	Increased	3.077	39
Gastrointestinal disease, hepatic system disease, organismal injury and abnormalities	Liver lesion	0.000048	Increased	2.594	53
Developmental disorder, embryonic development, organismal survival	Death of embryo	0.0000693	Increased	4.258	22
Cancer, organismal injury and abnormalities	Epithelial neoplasm	0.0000849	Increased	2.115	65
Cancer, organismal injury and abnormalities	Development of carcinoma	0.0000998	Increased	2.229	39
Cancer, organismal injury and abnormalities, respiratory disease	Development of lung carcinoma	0.000105	Increased	2.559	17
Cancer, organismal injury and abnormalities	Adenocarcinoma	0.000142	Increased	2.587	30
Cancer, cell death and survival, organismal injury and abnormalities	Cell death of tumor	0.000151	Increased	3.66	30
Cancer, cell death and survival, organismal injury and abnormalities, tumor morphology	Necrosis of tumor	0.000254	Increased	3.66	29
Cancer, organismal injury and abnormalities, respiratory disease	Lung adenocarcinoma	0.000393	Increased	2.375	15
Cancer, organismal injury and abnormalities, respiratory disease	Non-small cell lung carcinoma	0.000584	Increased	2.559	17
Cancer, organismal injury and abnormalities	Adenoma	0.00077	Increased	2.042	25
Developmental disorder, embryonic development	Degeneration of embryo	0.000814	Increased	2.804	8
Cancer, organismal injury and abnormalities	Carcinoma	0.00101	Increased	2.006	48
Developmental disorder, embryonic development, tissue morphology	Degeneration of embryoblast	0.00135	Increased	2.433	6
Cancer, cell death and survival, organismal injury and abnormalities, tumor morphology	Cell death of tumor cells	0.00151	Increased	3.536	26
Connective tissue disorders, developmental disorder, organismal injury and abnormalities, skeletal and muscular disorders	Dysplasia of skeleton	0.00204	Increased	2.2	7
Organismal survival	Perinatal death	0.00223	Increased	6.322	60
Developmental disorder, embryonic development, tissue morphology	Degeneration of embryonic tissue	0.00329	Increased	2.63	7
Neurological disease, organismal injury and abnormalities	Hydrocephalus	0.00329	Increased	3.138	11
Carbohydrate metabolism	Glycolysis of cells	0.00522	Increased	2	8
Lipid metabolism, molecular transport, small molecule biochemistry	Concentration of acylglycerol	0.00562	Increased	2.147	33
Cellular compromise	Dysfunction of mitochondria	0.00654	Increased	2.213	5
Cancer, cell death and survival, organismal injury and abnormalities, tumor morphology	Cell death of cancer cells	0.00661	Increased	3.252	20
Cancer, cell death and survival, organismal injury and abnormalities, tumor morphology	Cell death of osteosarcoma cells	0.00721	Increased	3.742	14
Cancer, organismal injury and abnormalities	Development of head and neck tumor	0.00724	Increased	2.189	11
Cellular assembly and organization, cellular function and maintenance	Organization of cytoskeleton	6.48E-08	Decreased	−3.001	101
Nervous system development and function, tissue morphology	Quantity of neurons	0.000000822	Decreased	−2.239	53
Cellular assembly and organization, cellular function and maintenance	Organization of cytoplasm	0.000000842	Decreased	−3.001	103
Cellular assembly and organization, cellular function and maintenance	Microtubule dynamics	0.00000115	Decreased	−3.268	86
Cell morphology, cellular assembly and organization, cellular function and maintenance	Formation of cellular protrusions	0.00000314	Decreased	−2.624	70
Cancer, organismal injury and abnormalities	Growth of tumor	0.00000351	Decreased	−3.22	75
Cellular movement	Cell movement	0.00000707	Decreased	−4.559	132
Tissue morphology	Quantity of cells	0.00001	Decreased	−3.306	171
Cellular growth and proliferation, connective tissue development and function, tissue development	Proliferation of connective tissue cells	0.0000108	Decreased	−2.856	49
Cellular movement	Migration of cells	0.0000139	Decreased	−4.26	117
Cellular movement, nervous system development and function	Migration of neurons	0.0000177	Decreased	−2.055	30
Connective tissue development and function, tissue development	Growth of connective tissue	0.0000256	Decreased	−2.686	50
Cellular assembly and organization	Quantity of intermediate filaments	0.000211	Decreased	−2	4
Nervous system development and function	Sensation	0.00041	Decreased	−2.482	30
Cancer, organismal injury and abnormalities	Neoplasia of tumor cell lines	0.000582	Decreased	−2.44	15
Cellular development, cellular growth and proliferation, nervous system development and function, tissue development	Development of neurons	0.000782	Decreased	−2.282	63
Cellular function and maintenance	Cellular homeostasis	0.000792	Decreased	−2.034	103
Organismal development	Size of animal	0.00102	Decreased	−2.322	22
Cell-to-cell signaling and interaction, nervous system development and function	Auditory evoked potential	0.00126	Decreased	−2.725	12
Behavior	Learning	0.00151	Decreased	−2.11	41
Lipid metabolism, small molecule biochemistry, vitamin and mineral metabolism	Synthesis of steroid hormone	0.00168	Decreased	−2.219	6
Tissue development	Formation of gland	0.00313	Decreased	−2.088	25
Embryonic development, organismal development	Development of body trunk	0.00377	Decreased	−3.116	95
Embryonic development, organ development, organismal development, skeletal and muscular system development and function, tissue development	Formation of muscle	0.00389	Decreased	−2.398	32
Cancer, organismal injury and abnormalities	Metastasis of tumor cell lines	0.00431	Decreased	−2.556	10
Organismal development	Development of genitourinary system	0.0049	Decreased	−3.43	86
Auditory and vestibular system development and function, nervous system development and function	Hearing	0.00506	Decreased	−2.157	15
Amino acid metabolism,post-translational modification, small molecule biochemistry	Phosphorylation of L-amino acid	0.00686	Decreased	−2	13
**Female ZF brain proteome analysis (HF/NF)**					
**Categories**	**Diseases or functions annotation**	***p*-value**	**Predicted activation state**	**Activation z-score**	**# Molecules**
Cellular assembly and organization, cellular function and maintenance	Organization of cytoskeleton	6.83E-08	Increased	2.675	101
Cellular assembly and organization, cellular function and maintenance	Organization of cytoplasm	0.000000884	Increased	2.675	103
Cellular assembly and organization, cellular function and maintenance	Microtubule dynamics	0.0000012	Increased	2.941	86
Cell morphology, cellular assembly and organization, cellular function and maintenance	Formation of cellular protrusions	0.00000326	Increased	2.483	70
Cancer, organismal injury and abnormalities	Growth of tumor	0.00000365	Increased	2.325	75
Cellular movement	Cell movement	0.00000746	Increased	2.14	132
Nucleic acid metabolism	Metabolism of nucleic acid component or derivative	0.000087	Increased	2.209	27
Cell morphology, cellular function and maintenance	Autophagy	0.000186	Increased	2.393	24
Nucleic acid metabolism, small molecule biochemistry	Metabolism of nucleotide	0.000212	Increased	2.209	23
Organismal survival	Viability	0.000296	Increased	3.302	14
Nervous system development and function	Sensation	0.000417	Increased	2.058	30
Cellular function and maintenance	Cellular homeostasis	0.000821	Increased	2.663	103
Organismal development	Size of animal	0.00104	Increased	2.322	22
Embryonic development, organismal development	Growth of embryo	0.00133	Increased	2.454	46
Cellular movement, embryonic development	Cell movement of embryonic cells	0.00172	Increased	2.2	12
Embryonic development, organismal development	Development of body trunk	0.00389	Increased	2.736	95
Cancer, organismal injury and abnormalities	Metastasis of tumor cell lines	0.00434	Increased	2.008	10
Nervous system development and function, tissue morphology	Quantity of neuroglia	0.00449	Increased	2.402	14
Nucleic acid metabolism, small molecule biochemistry	Synthesis of nucleotide	0.00583	Increased	2.019	15
Respiratory system development and function	Respiration of mice	0.00693	Increased	2	9
Embryonic development, organ development, organismal development, skeletal and muscular system development and function, tissue development	Development of striated muscle	0.00767	Increased	2.236	17
Organismal survival	Organismal death	2.59E-11	Decreased	−7.796	251
Developmental disorder, embryonic development, organismal survival	Death of embryo	0.0000704	Decreased	−2.198	22
Cancer, cell death and survival, organismal injury and abnormalities	Cell death of tumor	0.000153	Decreased	−2.141	30
Cancer, cell death and survival, organismal injury and abnormalities, tumor morphology	Necrosis of tumor	0.000258	Decreased	−2.141	29
Cancer, cell death and survival, organismal injury and abnormalities, tumor morphology	Cell death of tumor cells	0.00154	Decreased	−2.363	26
Connective tissue disorders, developmental disorder, organismal injury and abnormalities, skeletal and muscular disorders	Dysplasia of skeleton	0.00205	Decreased	−2.2	7
Organismal survival	Perinatal death	0.00228	Decreased	−3.588	60
Organismal survival	Death of perinatal stage organism	0.00619	Decreased	−2.137	11
Cellular compromise	Dysfunction of mitochondria	0.00657	Decreased	−2.213	5

### Protein Enrichment Analysis for Zebrafish Brain Proteome Induced by Acute Hypoxia and During Recovery

**FIGURE 2:**
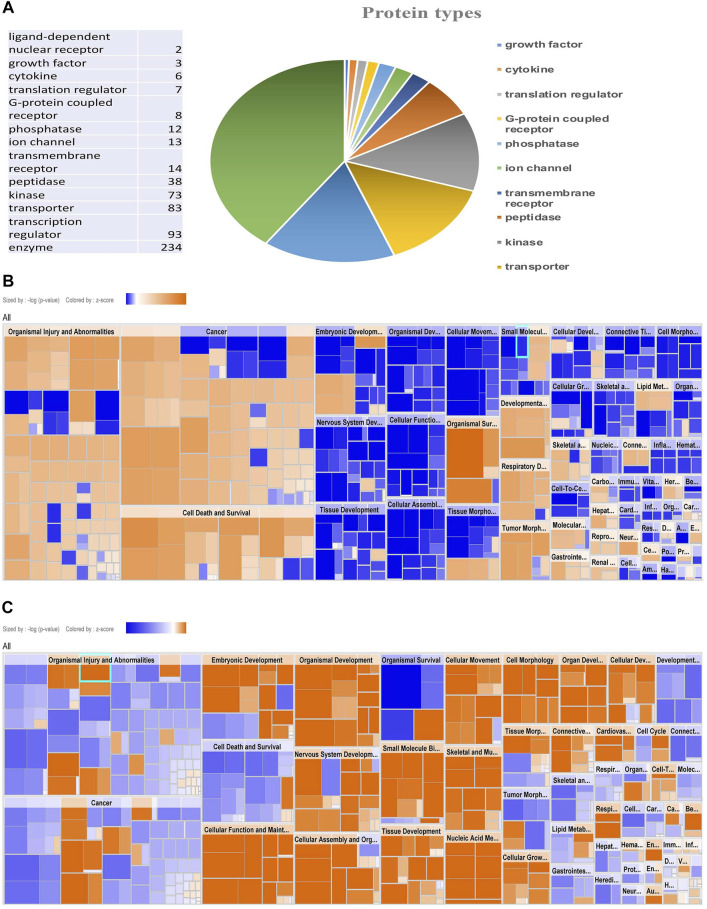
Protein enrichment analysis by IPA showing types of protein and Comparative heat map. Pie chart showing types of protein mapped by IPA **(A)**, Disease function Heat map of male **(B)** and female **(C)** zebrafish brain proteome upon hypoxia treatment.

Based on the zebrafish annotated database, IPA mapped 994 proteins out of 2,323 proteins identified in the iTRAQ analysis. These 994 proteins included different types of proteins i.e., transporters, transmembrane receptors, translation and transcription regulators, phosphatases, peptidase, kinases, enzymes, G-protein coupled receptors, ligand-binding receptors, and cytokines (Figure 2A). Out of all the groups, the majority of proteins belonged to the group “transcription regulators.”

The protein enrichment analysis on the altered proteome was performed using the Ingenuity Pathway Analysis (IPA) software. The disease function pathway-based heat map generated by the IPA (Figures 2B,C) clearly showed a sex-specific difference in the altered expression of proteins in different disease pathway conditions.

The IPA analysis for disease function annotation showed a predicted activation state with activation z-score and *p*-values and molecules involved in each category of disease function. Among all the 502 proteins mapped in IPA for the disease and function analysis, in males 65 categories of disease function showed the predicted activation state: 37 categories showed increased activation states while 28 categories exhibited decreased activation states. In females only 30 categories of disease function showed the predicted activation state and among those, 21 categories of disease function showed increased activation states and only 9 categories showed decreased activation states.

The most striking feature was the contrasting regulation between male and female in one of the disease and function categories named “organismal survival” and “leads to organismal death” with a very significant (*p* = 2.31E-11) activation z-score (12.434) identifying 251 molecules with an increased activation state, but the same 251 molecules showed a significantly (2.59E-11) decreased activation state with a z-score (−7.796) in females (Table 1). In males most of the increased activation state was observed in organismal injury, abnormalities, cell death, connective tissue disorders, developmental disorder, skeletal and muscular disorders, neurological, respiratory diseases, and cancer; whereas in females cellular assembly and organization, cellular function and maintenance, cell morphology, nucleic acid metabolism, and cellular movement showed an increased activation state. The disease function analysis clearly showed a sex-specific difference in hypoxia-induced neural damage and recovery as seen in our earlier studies (Das et al., 2019).

**FIGURE 3:**
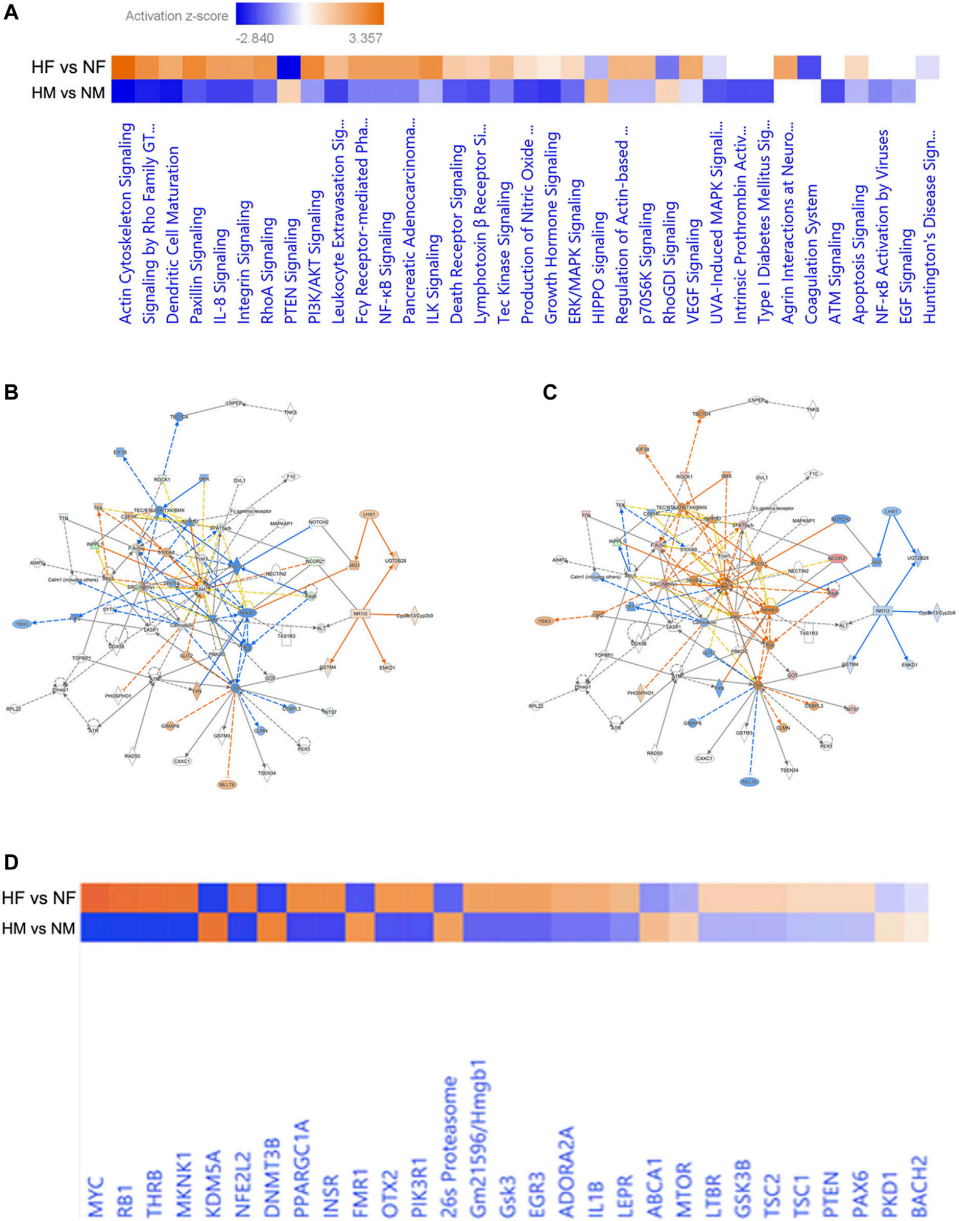
Pathway based on the top networks. Comparative heatmap for canonical pathways of male and female brain proteome induced by hypoxia **(A)**, Predictive pathway for zebrafish male **(B)** and female **(C)** brain proteome induced by hypoxia, comparative heatmap for upstream regulators **(D)**.

The IPA generated core expression analysis shed light on sex differences in canonical pathways with their predictive upregulation and downregulation in expression (Figure 3A). Among all, the top five canonical pathways were Actin Cytoskeleton Signaling, TCA Cycle II (Eukaryotic) 14-3-3-mediated Signaling, Remodeling of Epithelial Adherens Junctions, and Huntington’s Disease Signaling showed negative score in males while in females, a positive score was observed.

Hypoxia can induce cytoskeletal injury and remodeling through the activation of hypoxia-inducible factor-1α (HIF-1α) and HIF-1α activation results in actin cytoskeleton signaling (Weidemann et al., 2013; Huang et al., 2019). F-actin in non-muscle cells is to organize the actin cytoskeleton, which is utilized for cell locomotion, adhesion, and cell proliferation and we have observed activation of Factin in females (Figures 3B,C) indicating early proliferation in response to neural damage induced by hypoxia.

The core expression analysis of IPA led us to decipher many regulatory networks, disease function pathways, top upstream regulators and their predicted activation state, and also some biomarkers. Upon reviewing all the pathways involved, two individual pathways were found very interesting in males and females (Figures 3A,B), which clearly showed a gender-specific difference in the expression of a number of proteins in the pathway such as Rock1, Inppl1, Factin, Stat5ab, Ncor2, SRC-family, Got, Ints7 and Pdgfr, which were downregulated in the male brain but upregulated in the female brain. Though the pathways involved many molecules and networks, they were still centered around the AKT signaling pathway, which regulates a wide range of cellular functions and is involved in the resistance response to hypoxia-ischemia through the activation of proteins associated with cell survival, proliferation, and regulation of HIF-1α (Zhang et al., 2018). The growth factors and inflammation markers noticed in the pathway were studied in our previous study reported in (Das et al., 2019; Das et al., 2020).

The upstream regulators analyzed in IPA were 155 in male and 165 in female; among these 18 upstream regulators in male and 24 in female showed the predicted activation state (Table 2). Among the 18 upstream regulators in males, 14 were inhibited and only 4 were activated, and most of these were transcription regulators. Among the 24 upstream regulators in females, 16 were activated, with a majority of transcription regulators, and only with 8 were inhibited, which were not found in the males. Five upstream regulators [Myc, Mknk1, Nfe2l2 (Nrf2), Thrb, and otx2] were found to be common in both males and females with differential activation states, and interestingly these were in opposite directions, i.e., inhibited in males while activated in females.

**TABLE 2:** IPA generated upstream regulators analysis for male and female zebrafish brain proteome induced by hypoxia following recovery.

**Upstream regulators in male zebrafish brain with predicted activation state**											
**S. No.**	**Upstream regulator**	**Molecule type**		**Predicted activation state**		**Activation z-score**		***p*** **-value of overlap**		**Target molecules in dataset**	
1	MYC	Transcription regulator		Inhibited		−2.24		4.52E-05		BUB1, CCNA2, ENO1, EZH1, GLS, GOT1, GOT2, GPI, HIF1A, LDHB, MCM6, MCM7, PA2G4, PDHA1, PDK1, PGAM1, PGK1, PKM, TCF3	
2	RB1	Transcription regulator		Inhibited		−3.183		0.000472		ACO2, Actn3, APAF1, ATP1A1, ATP5F1A, BCKDHA, CASP9, CCNA2, CKM, DLST, FABP2, HSBP1, KRT18, MCM7, MFN2, MLYCD, MYH4, MYH6, MYH7, NDUFB10, RBL1, TIMM22	
3	TSC2	Other		Inhibited		−2.433		0.000695		IRS1, IRS2, MCL1, PDGFRB, PRKCA, PSMC3	
4	CSF2	Cytokine		Inhibited		−3.141		0.00347		BUB1, C3, CCNA2, CHTF18, E2F8, EXO1, FBXO5, FIGNL1, IL12B, KNTC1, MCM6, MNS1, NOS2, POLD1, POLE, SMC2, TLR4	
5	MKNK1	Kinase		Inhibited		−3.317		0.00792		APC, ATP1A3, CRMP1, DPYSL3, HADHA, KIF5A, MYO6, PRKAR1B, SNAP25, STXBP1, THRA	
6	Gsk3	Group		Inhibited		−2.219		0.00826		COL2A1, KDR, MYH6, NOS2, STAT1	
7	NFE2L2	Transcription regulator		Inhibited		−2.066		0.00931		ACTG1, ALAS2, APOA4, ATP1A1, CDKN2C, ESD, FKBP5, G6PD, GFAP, GSTP1, HMOX1, L1CAM, MCFD2, NCKAP1, NOS2, NQO1, PDIA3, PFN2, PSMC1, PSMC3, PSMD11, RAN, SCG2, SREBF1, SYP, TTR, VCP	
8	THRB	Ligand-dependent nuclear receptor		Inhibited		−3.054		0.0121		ABCD3, CSHL1, DDC, DIO1, FGFR3, IGFBP2, MAPK8, MYH6, MYH7, NCOR2, STAT5B, WNT4, YWHAE	
9	LEPR	Transmembrane receptor		Inhibited		−2.791		0.0139		APOA1, APOA4, CREB3L2, CSHL1, EXOC4, GFAP, HIF1A, INPPL1, IRS2, MMP14, NBN, PLCB3, SNAP25, SREBF1	
10	IL1B	Cytokine		Inhibited		−2.59		0.0182		A2M, ATP1A1, C3, COL2A1, FKBP5, FOXO1, HAS2, HIF1A, KIF15, MMP9, NOS2, STAT1	
11	EGFR	Kinase		Inhibited		−2.314		0.0258		ACY1, ATAD3A, CCNA2, CCT5, GFAP, HAS2, MMP14, MMP9, PA2G4, TUBA4A, UBA1	
12	OTX2	Transcription regulator		Inhibited		−2.219		0.0435		A2M, EN1, PRDM1, SIX3, TF, TTR	
13	UCHL1	Peptidase		Inhibited		−2		0.366		ANXA6, LDHB, MAPK6, SCP2	
14	STAT6	Transcription regulator		Inhibited		−2.433		1		BCL6, Cmah, IL12B, IRS2, MMP14, MMP9, MYO6, NCOA3, SERPINA1	
15	KDM5A	Transcription regulator		Activated		2.688		0.00249		ACO2, Actn3, ATP1A1, ATP5F1A, BCKDHA, DLST, HSBP1, MFN2, MLYCD, MYH4, MYH6, MYH7, NDUFB10, TIMM22	
16	26s Proteasome	Complex		Activated		2.236		0.00826		APAF1, BHLHE22, FOXO1, NOTCH1, PRKCA	
17	HAND1	Transcription regulator		Activated		2		0.0179		KDR, MLYCD, NOTCH1, NRP1	
18	SATB1	Transcription regulator		Activated		2		0.472		APC, ETS1, NCOR1, NR2C2	
**Upstream regulators in female zebrafish brain with predicted activation state**											
**S. No.**	**Upstream regulator**	**Molecule type**		**Predicted activation state**		**Activation z-score**		***p*** **-value of overlap**		**Target molecules in dataset**	
1	MYC	Transcription regulator		Activated		2.801		4.58E-05		BUB1, CCNA2, ENO1, EZH1, GLS, GOT1, GOT2, GPI, HIF1A, LDHB, MCM6, MCM7, PA2G4, PDHA1, PDK1, PGAM1, PGK1, PKM, TCF3	
2	INSR	Kinase		Activated		2.124		0.000466		ACO2, ACTA1, ACTN4, ALDH6A1, ATP5F1A, ATP5F1B, CS, DCTN4, FLNC, GOT2, HADHA, HSPD1, IDH3A, IGF2R, INSR, MDH2, MMP9, MPEG1, MYH7, NAMPT, OGDH, PDHA1, PDHB, PKLR, SCP2, SREBF1	
3	Gm21596/Hmgb1	Transcription regulator		Activated		2.219		0.00101		HIF1A, NOS2, PKM, SIGIRR, TLR4	
4	PIK3R1	Kinase		Activated		2.621		0.00711		FOXO1, HIF1A, HMOX1, IL12B, NOS2, PDHA1, PDK1, PKM	
5	MKNK1	Kinase		Activated		2.111		0.00798		APC, ATP1A3, CRMP1, DPYSL3, HADHA, KIF5A, MYO6, PRKAR1B, SNAP25, STXBP1, THRA	
6	NFE2L2	Transcription regulator		Activated		2.705		0.00943		ACTG1, ALAS2, APOA4, ATP1A1, CDKN2C, ESD, FKBP5, G6PD, GFAP, GSTP1, HMOX1, L1CAM, MCFD2, NCKAP1, NOS2, NQO1, PDIA3, PFN2, PSMC1, PSMC3, PSMD11, RAN, SCG2, SREBF1, SYP, TTR, VCP	
7	EGR3	Transcription regulator		Activated		2.219		0.0116		BCL6, ESD, LMO7, NOTCH1, PABPC1L	
8	THRB	Ligand-dependent nuclear receptor		Activated		3.054		0.0122		ABCD3, CSHL1, DDC, DIO1, FGFR3, IGFBP2, MAPK8, MYH6, MYH7, NCOR2, STAT5B, WNT4, YWHAE	
9	MYB	Transcription regulator		Activated		2		0.019		CLTA, HSPA8, MAD1L1, NOTCH1, RGS8, SLC27A2, TULP4	
10	OTX2	Transcription regulator		Activated		2.219		0.0437		A2M, EN1, PRDM1, SIX3, TF, TTR	
11	SRF	Transcription regulator		Activated		2.957		0.0551		ACTA1, ACTG1, CKM, ETS1, ITGA2B, ITGB1, KDR, MYH4, MYH6, MYH7, PRSS57, TTN, VCL	
12	HIF1A	Transcription regulator		Activated		2.008		0.165		ENO1, IL12B, MIF, NOS2, NOTCH1, PDHA1, PDK1, PKM, TTN	
13	HOXD10	Transcription regulator		Activated		2		0.193		DAPP1, RSAD2, TFR2, WDR5	
14	Creb	Group		Activated		2		0.296		ARHGEF9, CBWD1, GABBR1, GRK3, INTS7, MCL1, PGK1, POLE, PRKCA	
15	HNF4A	Transcription regulator		Activated		2.382		0.329		CCNA2, ELMO1, FABP2, HIF1A, HSPA8, KRT8, NBEA, PFN2, PKM, RSPH4A, SCP2, SERPINA1, TF, TFR2, WNT4	
16	mir-223	MicroRNA		Activated		2		1		ALCAM, CRHBP, NQO1, TLR4	
17	DNMT3B	Enzyme		Inhibited		−2.53		0.00754		ACTA1, CKM, GRK3, KDR, MYH6, MYH7, PIK3C2B, PRKAR1B, SLC8A2, STAT1	
18	MAT1A	Enzyme		Inhibited		−2		0.0149		APOA1, KRT18, MAT1A, PRDX6	
19	CHADL	Other		Inhibited		−2		0.061		COL2A1, CSPG4, MN1, NTRK3	
20	PTPN1	Phosphatase		Inhibited		−2.219		0.0762		HHEX, IRS1, IRS2, NOS2, TMEM26	
21	DNMT3A	Enzyme		Inhibited		−2.121		0.142		ACTA1, CKM, GRK3, IRS1, MYH6, MYH7, PIK3C2B, SLC8A2	
22	ZNF106	Other		Inhibited		−2		0.204		ALAS2, C3, NDUFB10, PRDX2	
23	BTNL2	Transmembrane receptor		Inhibited		−2		0.55		CDKN2C, DAPP1, NTRK3, S100A4	
24	DICER1	Enzyme		Inhibited		−2.138		1		CCNG1, CRH, ERBB2, HNRNPH1, IGF2R, ITGB1, MMP9, NOTCH1, PRKCA	

In Table 3 the regulator effects of males and females are shown, where only one of the regulators, Nfe2l2, is common in both, but with only one common target molecule VCP (valosin containing protein), and all different target molecules in both the dataset VCP was earlier reported to be an AKT binding protein, and its expression was found enhanced in hypoxia (Klein et al., 2005).

**TABLE 3:** IPA generated regulatory effect analysis for male and female zebrafish brain proteome induced by hypoxia following recovery.

Male hypoxia regulator effects								
ID	Consistency score	Regulators		Target total	Target molecules in dataset		Diseases & functions	Known regulator-disease/function relationship
1	2.309	HAND1,THRB		12	CSHL1,FGFR3,IGFBP2,KDR,MAPK8,MLYCD,MYH6,NCOR2,NOTCH1,NRP1,STAT5B,WNT4		Cellular homeostasis, development of body trunk, development of genitourinary system	67% (4/6)
2	2.111	MYC		11	BUB1,CCNA2,ENO1,GLS,GPI,HIF1A,PA2G4,PDHA1,PDK1,PKM,TCF3		Carcinoma, frequency of tumor, growth of tumor, incidence of tumor	100% (4/4)
3	2	NFE2L2		4	PSMC1,PSMD11,RAN,VCP		Cell death of tumor cells	0% (0/1)
4	1.789	OTX2		5	A2M,EN1,PRDM1,SIX3,TF		Quantity of cells	100% (1/1)
5	−5.715	MYC		6	ENO1,GPI,HIF1A,PDK1,PGK1,PKM		Glycolysis of cells	100% (1/1)
6	−7.506	HAND1		3	KDR,NOTCH1,NRP1		Migration of cells	0% (0/1)
7	−16.743	HAND1		3	KDR,NOTCH1,NRP1		Organization of cytoplasm	0% (0/1)
8	−19.23	MYC		5	BUB1,CCNA2,GPI,HIF1A,PDK1		Growth of connective tissue	100% (1/1)
**Female hypoxia regulator effects**								
**ID**	**Consistency score**		**Regulators**	**Target total**		**Target molecules in dataset**	**Diseases & functions**	**Known regulator-disease/function relationship**
1	3.051		Gm21596/Hmgb1,PIK3R1,THRB	13		CSHL1,FGFR3,FOXO1,HIF1A,HMOX1,IL12B,MAPK8,MYH6,NCOR2,NOS2,STAT5B,TLR4,WNT4	Autophagy, development of body trunk	50% (3/6)
2	−4.082		MKNK1	6		ATP1A3,HADHA,KIF5A,SNAP25,STXBP1,THRA	Perinatal death	0% (0/1)
3	−4.491		PIK3R1	6		FOXO1,HIF1A,HMOX1,IL12B,NOS2,PDK1	Cell movement	100% (1/1)
4	−5.367		MKNK1	5		APC,CRMP1,DPYSL3,KIF5A,MYO6	Microtubule dynamics	0% (0/1)
5	−6.5		Gm21596/Hmgb1	4		HIF1A,NOS2,PKM,TLR4	Growth of tumor	100% (1/1)
6	−7.5		NFE2L2	4		G6PD,NOS2,NQO1,VCP	Metabolism of nucleotide	0% (0/1)

### Validation of Few Selected Regulator Effects Molecules From IPA Analysis

**FIGURE 4:**
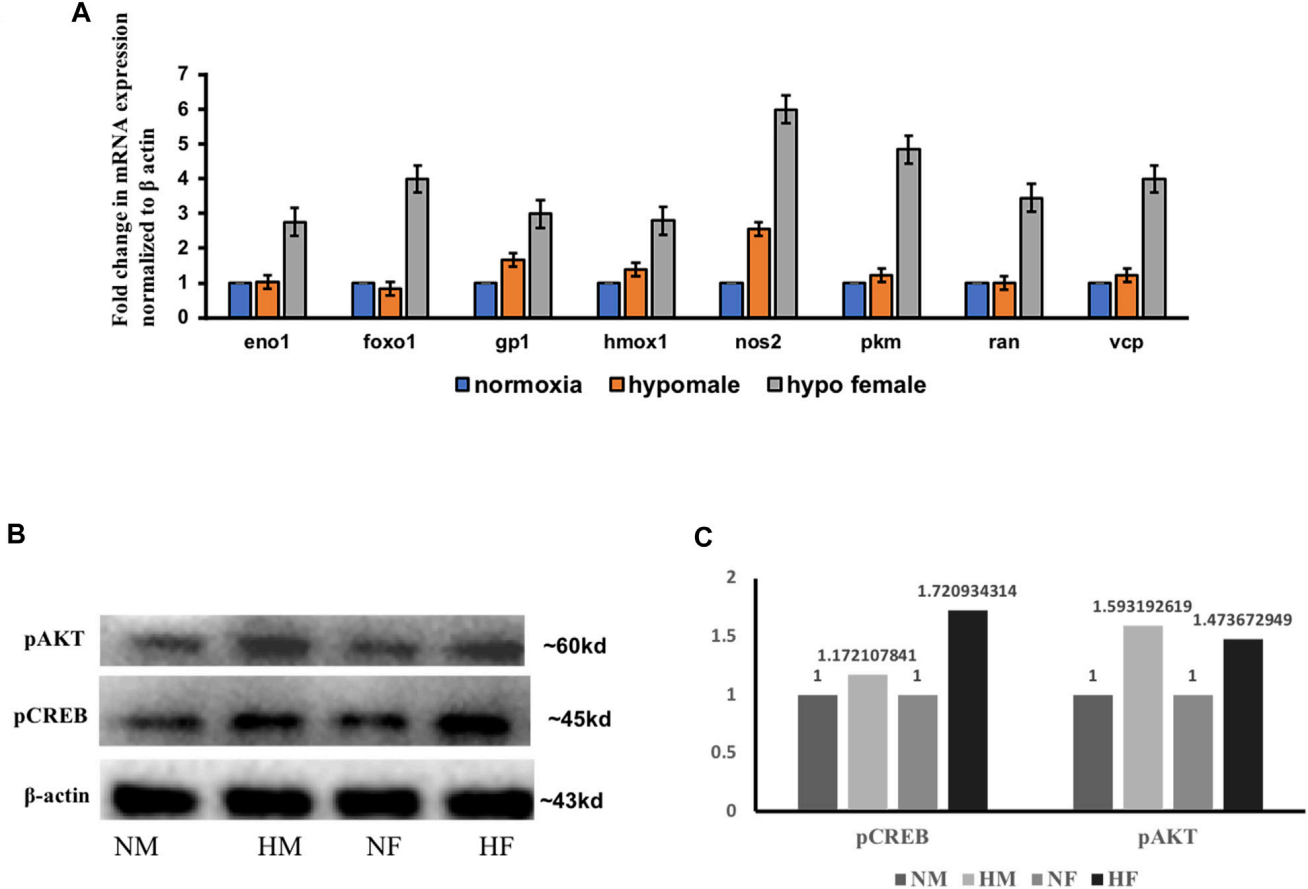
Validation of few regulatory target molecules. Graph showing mRNA expression of eno1, foxo1, gp1, hmox1, nos2, pkm, ran, and vcp **(A)** The data are expressed as the mean ± SEM, (*n* = 6 pooled brains). Immunoblot showing expression of pCREB and pAKT in male and female brain **(B)**, Densitometry for comparison of protein expression in HM vs. NM and HF vs. NF **(C)**.

A few of the target molecules (Eno1, Foxo1, Gp1, Hmox1, Nos2, Pkm, Ran, and Vcp) from both the data sets were considered for validation by quantitative Realtime PCR (Figure 4). eno1 (enolase 1) is one of the HIF target genes (Benita et al., 2009). The qPCR analysis revealed more than ∼2-fold increase in eno1 and ran (ras-related nuclear protein) in females but it remained unchanged in males. The foxo transcriptional factors are important regulators of cell survival in response to various stresses including oxidative stress (Bakker et al., 2007). foxo1 was upregulated ∼4-fold in females but unaffected in males thus indicating better survival response after hypoxia in females. The expression of gp1 (Glycoprotein 1) and hmox1 (Heme Oxygenase 1) showed a similar kind of expression pattern in both sexes, a mild upregulation in males, and ∼3-fold upregulation in females. (gp1) acts as a glycolytic enzyme, as well as functioning as a tumor-secreted cytokine and an angiogenic factor (AMF) that stimulates endothelial cell motility, GPI is also a neurotrophic factor (Neuroleukin) for spinal and sensory neurons. The role of neurotrophic factors in repair mechanisms are well evident, therefore in our study 4 h post-hypoxia females are better in recovery speed as compared to males. Hmox1 has been shown to be induced by various stresses including hypoxia (Panchenko et al., 2000); our study also revealed an increase in its expression. The expression of nos2 (nitric oxide synthase) and pkm (pyruvate kinase M) is inducible with hypoxia and hif1 targets showed a higher fold upregulation in females as compared to males.

Various mediators like growth factors, glucose transporters, solute carriers, neurotransmitters, inflammatory molecules, and stress signals as well as factors known to modulate the intracellular cAMP or Ca^2+^ levels can activate cAMP-responsive element-binding protein (CREB) through phosphorylation of serine 133 (Ser^133^) by protein kinase A (PKA) and protein kinase B (PKB/AKT) (Steven et al., 2016). The pathway generated by IPA in Figures 3A,B was also centered on the AKT pathway with many downstream interacting molecules involved in cell death and repair mechanisms. Among all the molecules, we evaluated the expression of two of the major molecules in acute hypoxia recovery with respect to sex difference and found a sex difference in the expression of PCREB and PAKT (Figures 4B,C) where in females activation of CREB and AKT leads to early cell death survival and repair.

### Analysis of Proteins Showing Uniform (Either Upregulated or Downregulated) Expression in Both the Sexes

Throughout the protein enrichment analysis by IPA sex-specific, global proteome changes in the acute hypoxia zebrafish model were observed, which is in concurrence with our previous study (Das et al., 2019). But the question which remains unsolved is why the recovery in females is quicker than in males. At 4 h post-hypoxia when both the sexes survived coping up with the neural damage then there must be some common mechanism involved for recovery. So rather than looking further into the differentially expressed markers, we looked into the shared regulation of proteins. In Figure 1B we have shown the analysis of proteins resulted from iTRAQ where 554 proteins have a common expression pattern in both the sexes and among them 478 proteins were found regulated in one direction i.e., upregulated in both male and female brain in response to acute hypoxia. We hypothesized that as animals from both sexes are in the recovery process, therefore, a common mechanism of regulation may help to elucidate the mechanism behind the later recovery of the males from neural damage induced by hypoxia-ischemia. While looking into the 478 upregulated proteins, among the top five upregulated proteins in males, we identified histone-lysine N-methyltransferase H3 lysine-9 specific 5 protein, an epigenetic regulator displaying ∼3-fold upregulation in the male brains and ∼1.6-fold upregulation in the female brains (Table 4). Post-translational modifications of histones are widely recognized as an important epigenetic mechanism in the organization of chromosomal domains and gene regulation. Methylation of lysine 4 and acetylation of lysine 9 of histone H3 has been associated with regions of active transcription, whereas methylation of H3K9 and H3K27 are generally associated with gene repression (Litt et al., 2001; Nakayama et al., 2001; Maison et al., 2002; Peters et al., 2002; Vakoc et al., 2006). Recently, hypoxia-induced histone modifications in neural gene regulation have been reported, and these were found on both hypoxia-activated and hypoxia-repressed genes (Johnson et al., 2008). H3K9 methylation is a critical epigenetic mark for gene repression and silencing. Hypoxia induces H3K9 methylation at different gene promoters, which is correlated with the repression and silencing of those genes following hypoxia (Lu et al., 2011).

**TABLE 4:** Top 5 upregulated proteins retrieved from uniformly regulated (upregulated) in both male and female zebrafish brain induced by acute hypoxia.

**S. No.**	**Accession No.**	**Description**	**HM/NM**	**HF/NF**
1	56693350	Very long-chain acyl-CoA synthetase	3.234	1.690
**2**	**71834420**	**Histone-lysine N-methyltransferase, H3 lysine-9 specific 5**	**3.188**	**1.603**
3	189531944	Predicted: hypothetical protein LOC100148665	3.057	1.966
4	326666355	Predicted: zinc finger protein 208-like	2.984	2.606
5	326664965	Predicted: protein FAM5C	2.742	3.703

H3K9 which was the focus of manuscript and taken for further mechanistic analysis so made it bold.

### Deciphering the Role of H3K9me3 by Co-Immunoprecipitation and ChIP qPCR

**FIGURE 5:**
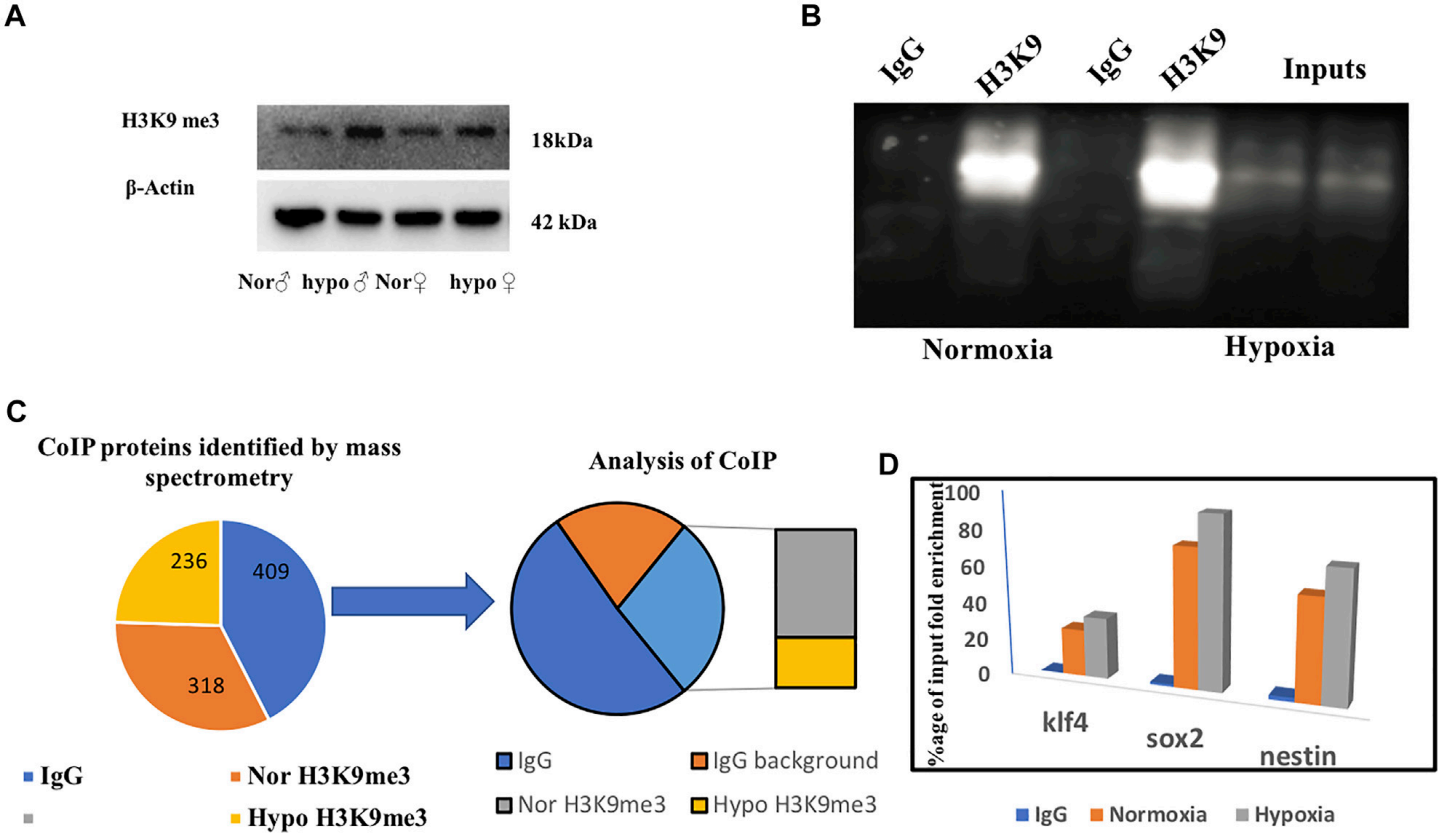
Deciphering the role of H3K9me3 by co-immunoprecipitation. Immunoblot of H3K9me3 showing upregulation in hypoxia **(A)**, CoIP of H3K9me3 in zebrafish male brain nuclear protein **(B)**, Schematic representation of CoIP analysis **(C)**. ChIP qPCR showing H3K9 occupancy on target gene promoter. ChIP qPCR showing the increase in H3K9me3 enrichment on the promoter region of klf4, sox2, and nestin in hypoxic male brain **(D)**.

Based on the previous literature (Lindeman et al., 2010), we hypothesized that H3K9 can be our prime target for deciphering late recovery in males as it was significantly upregulated in the male brain following hypoxia and being a repressive epigenetic mark in nature its high level can repress and/or silence a number of critical neural genes. Considering the role of H3K9me3 in hypoxia (Chakravarty et al., 2016) we immunoblotted for H3K9me3 using a specific antibody and performed a co-immunoprecipitation (CoIP) to identify the interacting proteins of H3K9me3 in hypoxic condition (Figures 5A–C). We could validate the expression of H3K9me3 through immunoblotting with an upregulation of H3K9me3 in hypoxia males when compared to normoxia males (Figure 5A). For CoIP experiment, nuclear extract was isolated from male zebrafish brains. The eluted proteins were then detected for immunoprecipitated and co-immunoprecipitated proteins by SDS-PAGE followed by western blotting. Then, 5% of the initial lysates were used as the input (Figure 5B). A mass spectrometric approach was used to identify the co-immunoprecipitated proteins obtained by the pull-down of the target antibody. The resultant peptides from MS/MS for four groups (normoxia IgG, normoxia and hypoxia H3k9me3 pull-down) were analyzed and after removing the background of IgG pooled proteins we could obtain 153 proteins identified in male normoxia H3K9me3 pull-down and 72 proteins identified in male hypoxia H3K9me3 pull down. Surprisingly, there were no common proteins in the normoxia and hypoxia H3K9me3 pull-downs, showing hypoxia stress may lead to alteration in interacting proteins. Further, we went through our iTRAQ data and tried to see whether these co-immunoprecipitated proteins were also found altered post-hypoxia in our high throughput proteomics data where almost all the proteins were found to overlap (Figure 5C).

The co-interacted proteins of H3K9me3 could not answer the unresolved question of why the males are recovering later. Therefore, we thought of evaluating the transcriptional targets of H3K9me3 to get an answer to our question, as H3K9me3 is a repressive marker so its upregulation in males may repress any neurogenic marker needed for recovery from hypoxia-induced neural damage. An earlier report on chromatin state of the developmentally regulated genes (Lindeman et al., 2010) led us to explore the striking upregulation of transcriptionally repressive epigenetic marker H3K9me3 at 4 h post hypoxia in zebrafish male brain.

**FIGURE 6:**
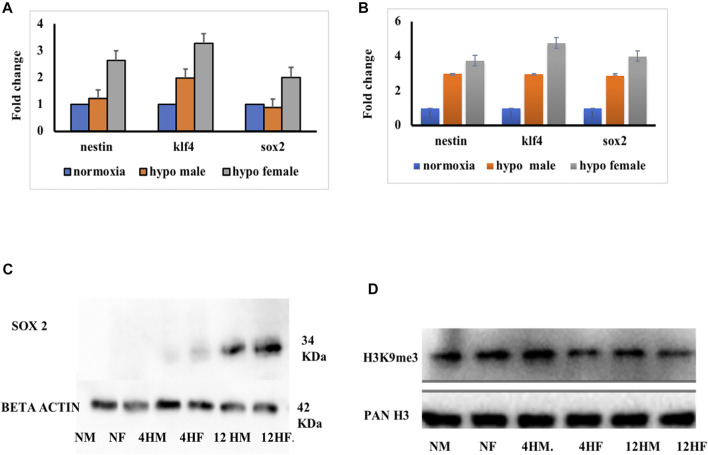
Validation ChIP analysis. mRNA expression at 4 h **(A)** and 10 h **(B)** post hypoxia in both male and female (*n* = pooled 6 brain). Expression of sox2 protein at different times of recovery from hypoxia **(C)**. Expression of H3K9me3 at different times of recovery from hypoxia **(D)**.

The ChIP-qPCR data showed the repression of early neurogenesis markers nestin, klf4, and sox2 in the zebrafish male brain 4 h post-hypoxia (Figure 5D). It is pertinent to mention here that the ChIP assay was not performed on female zebrafish brains as the male brain proteome showed a higher fold upregulation in H3K9me3. For further validating the data the mRNA expression levels for nestin, klf4, and sox2 at two-time points of recovery i.e., at 4 h (Figure 6A) and 12 h (Figure 6B) post hypoxia, was assessed. The qPCR analysis showed at 4 h post-hypoxia the expression of early neurogenic markers showed mild activation in males and later at 12 h post-hypoxia the expression was much higher. The protein level expression of Sox2 was evaluated at low concentration (25 μg) of protein which showed in both males and females at 4 h post-hypoxia but the expression was quite low in both the sexes; in the male it was almost negligible, however in female a mild expression was observed, which at the later time point i.e., 12 h post-hypoxia showed noticeable upregulation in both male and female brain (Figure 6C).

To further validate if the sox2 expression is dependent on H3K9me3 level, the expression level of H3K9me3 was assessed and predictably it was found upregulated in the male brain as shown in the previous experiment, compared to the female brain at 4 h post-hypoxia. Later at 12 h post-hypoxia, the level of H3K9me3 was much less in males than what it was at 4 h post-hypoxia (Figure 6D). This result suggested that with the activation of H3K9me3 the expression levels of early neurogenic markers are getting repressed. This could be the possible reason for late recovery in males as early neurogenic markers are not fully activated in response to hypoxia insult, in contrast to the female brain.

Among Cerebral strokes, ischemic stroke is the most common type of stroke and a major cause of death and/or disability worldwide, though there are continuous efforts to establish a proper diagnosis and efficient therapy. The proteomics study complements both genomics and transcriptomics and simultaneously provides information about the proteins that can be implemented for main functional mediators of cells such as their post-translational modification and their interactions with biological molecules. However, post-stroke is mostly related to protein function which can be a direct target for therapeutic intervention. Therefore in the present study, we performed a quantitative proteomics approach for hypoxia-induced brain to identify favorable biomarkers involved in neuronal injury and recovery (Li et al., 2019). In our previous study, clear sex-specific differences were observed in acute hypoxia-induced neural damage and recovery but to explore more about the mechanism of recovery in the present study we have focused on a 4 h post hypoxia timepoint, predicting this could possibly be a viable therapeutic window. To date, many high throughput studies on hypoxia (Durukan and Tatlisumak, 2007; Cuadrado et al., 2010; Goldenberg et al., 2014; Chen et al., 2015; Durukan and Tatlisumak, 2007; Cuadrado et al., 2010; Goldenberg et al., 2014; Chen et al., 2015; Ton et al., 2003; van der Meer et al., 20052005; Shah et al., 2019) gave sufficient information about the genes and proteins involved in hypoxia and related diseases but the roles of these hallmarked hypoxia markers are not well studied in a sex-specific context. Therefore, we have attempted to emphasize more on the sex-specific neural regulation post-hypoxia, which will provide a better insight into designing efficient therapeutics for patients who suffered acute hypoxic insults. The prevalence of hypoxic brain damage is increasing and prognostic factors for either poor or good outcome are lacking (Heinz and Rollnik, 2015).

The advantage over traditional proteomics and iTRAQ based proteomics is that in iTRAQ all four groups can be simultaneously processed to reduce the error rate and post-translational modifications can also be quantified. The present study on whole zebrafish brain proteome upon global acute hypoxia sheds light on many differential roles of protein markers which can be further validated. Solute carrier (SLC) transporters are well-known therapeutic targets (Lin et al., 2015) and in our study too we have observed a very high activation in recovery. We tried to elucidate the role of one of the histone-based epigenetic regulatory mechanism (H3K9me3) that controls adult neurogenesis during the recovery phase post-hypoxia-ischemia. There is hardly any study on the epigenetic mechanisms in the zebrafish brain to date. Here, we identified hundreds of transcription factors involved in post-hypoxia recovery in a gender-specific manner, which can add to the development of a better therapeutic strategy.

## Conclusion

To conclude we have studied the sex-specific difference in global proteome changes in zebrafish brain induced by acute hypoxia and during the recovery. We elucidated the unresolved question from our previous study (Das et al., 2019) regarding the delayed recovery in males following hypoxic insult. With the striking upregulation of H3K9me3 in males at 4 h post-hypoxia, the early neurogenic markers like nestin, klf4, and sox2 expression level got affected, which might be the reason for late recovery in males, compared to females. Acute hypoxia-induced sex-specific comparison of brain proteome led us to reveal many differentially expressed proteins including the novel ones, which can be further studied for the development of novel targets and a better therapeutic strategy.

## Data Availability

The datasets presented in this study can be found in online repositories. The names of the repository/repositories and accession number(s) can be found below: http://proteomecentral.proteomexchange.org/, PXD027528.
